# Acute knee extensor torque and muscle oxygenation responses to isometric testing in exercise and non‐exercised legs of male adults

**DOI:** 10.14814/phy2.70680

**Published:** 2025-12-01

**Authors:** Carlos Sendra‐Pérez, Joaquín Martín Marzano‐Felisatti, Willian da Silva, Pedro Pérez‐Soriano, Felipe P. Carpes, Jose Ignacio Priego‐Quesada

**Affiliations:** ^1^ Research Group in Sports Biomechanics (GIBD), Department of Physical Education and Sports Universitat de València Valencia Spain; ^2^ Escuela de Kinesiología, Facultad de Ciencias Pontificia Universidad Católica de Valparaíso Valparaíso Chile; ^3^ Applied Neuromechanics Group, Laboratory of Neuromechanics Federal University of Pampa Uruguaiana Brazil

**Keywords:** fatigue, near‐infrared spectroscopy, physical exercise, recovery, testing

## Abstract

Portable near‐infrared spectroscopy (NIRS) provides a noninvasive measure of local muscle metabolic demand through muscle oxygen saturation (SmO_2_). This study aimed to evaluate acute SmO_2_ responses in exercising and non‐exercising muscles during an isometric test conducted before and after exercise. Twelve physically active men (25 ± 6 years; 1.75 ± 0.07 m) performed three maximal voluntary contractions (MVC) and a 45‐s isometric contraction at 70% MVC (IC70%) before and after completing five sets of 30 concentric knee extensions at 180°·s^−1^, with 60 s rest between sets, on an isokinetic dynamometer. SmO_2_ was recorded in the vastus lateralis (exercised and non‐exercised leg) and triceps brachii. Significant differences in SmO_2_ were observed between exercised and non‐exercised legs both pre‐ (*p* = 0.048) and post‐exercise (*p* < 0.001), whereas no significant changes were found in the triceps brachii or non‐exercising vastus lateralis. Acute responses to isometric exercise differed between SmO_2_ and torque measures. While the torque of the IC70% did not change following the exercise protocol, SmO_2_ showed a higher deoxygenation response during exercise. The isometric test did not indicate any redistribution of muscle oxygenation from non‐exercising to exercising muscles. These results provide novel insights into the dissociation between mechanical and microvascular responses during isometric exercise and support the potential of this test as a practical tool for monitoring training loads.

## 1 INTRODUCTION

Oxygen plays a crucial role in metabolism during muscle activity, particularly for mitochondrial respiration. The oxidation process produces adenosine triphosphate (ATP) (Nolfi‐Donegan et al., 2020), and without sufficient oxygen, the ATP production process becomes less efficient, contributing to muscle fatigue (Kime et al., 2003; Perrey et al., 2024). Since peripheral fatigue mainly relates to disturbances in the surface membrane (i.e., due to an increased [K^+^] in venous) and metabolic factors associated with oxygen supply (Fitts, 1994), there is growing interest in using portable near‐infrared spectroscopy (NIRS) technology to assess muscle fatigue through the microvascular responses detected by oxygen availability (Iannetta et al., 2019; Perrey et al., 2024). Assessing fatigue in sports science is crucial for the proper planning of training workloads aimed at optimal performance in competition and reduced injury risk (Thorpe et al., 2017).

NIRS is a technology that assesses microvascular responses by monitoring changes in hemoglobin ([Hb]) and myoglobin ([Mb]) oxygen levels. This is achieved through the detection of light‐absorbing chromophores in biological tissues (Barstow, 2019). Muscle oxygen saturation (SmO_2_), which represents the ratio of deoxygenated to oxygenated [Hb] or [Mb], is a key measure derived from this technology (Barstow, 2019; Perrey et al., 2024). Recent work has demonstrated that NIRS can effectively capture the kinetics of oxygen extraction and reoxygenation during and after exercise, providing insight into both local perfusion and oxidative metabolism (Iannetta et al., 2019; Rosenberry et al., 2018). In particular, Iannetta et al. (2019) showed that muscle oxygenation kinetics reflect the balance between oxygen delivery and utilization at the microvascular level, emphasizing the sensitivity of NIRS‐derived SmO_2_ to changes in exercise intensity. Similarly, Rosenberry et al. (2018) highlighted the potential of NIRS to detect microvascular adjustments across different occlusion protocols. Although previous studies have evaluated the effects of exercise on microvascular responses during several occlusion tests (Dominelli et al., 2024; Greaves et al., 2024; Keller et al., 2023; Shoemaker et al., 2024) (e.g., approximately 5 min at 50 mmHg above systolic pressure for the arm and 100 mmHg above systolic pressure for the leg) (Barstow, 2019), these kinds of occlusion tests are difficult to apply in field settings and across different populations. To date, only a few studies have provided an applied context for measuring the workload in the skeletal muscle through SmO_2_ during resistance training, specifically during back squat exercise (Chan et al., 2025; Gómez‐Carmona et al., 2020). Moreover, there is a clear lack of studies employing specific protocols to measure acute responses to exercise using microvascular responses after exercise at varying intensities, whether strength or endurance. Furthermore, before verifying whether there are microvascular responses to acute fatigue, it is important to verify whether responses are also produced by intensity exercise per se, since this would increase the difficulty of the specific protocol in differentiating between the acute effects of exercise. In this sense, recent research has emphasized that microvascular responses to exercise are not limited to the working muscles (Miranda‐Fuentes et al., 2021), making it relevant to also examine responses in non‐exercising muscles and use them as a control region to identify the specific adaptations occurring in the active muscles.

An incremental protocol in cycling might be useful for evaluating SmO_2_ responses to exercise in different muscles, as demonstrated by some studies (Sendra‐Pérez et al., 2025; Yogev et al., 2022). Yogev et al. (2022) compared oxygenation responses of non‐locomotor and locomotor muscles during cycling exercise. They found that the deltoid muscle experienced increased O_2_ extraction due to a redistribution of blood flow primarily towards the working muscles (Yogev et al., 2022). In addition to dynamic settings, isometric exercises have been used in both upper limbs (Scano et al., 2020) and lower limbs (Moalla et al., 2006; Yamada et al., 2008) for exercise monitoring using measurements of SmO_2_. Indeed, a previous study found a high correlation between NIRS outcomes (i.e., SmO_2_ and [Hb]) and electromyography parameters, suggesting that NIRS could be useful as a biomarker of acute adaptations (Scano et al., 2020). Therefore, establishing an isometric exercise protocol to measure acute exercise responses through muscle oxygenation could be a quick and easy method to monitor responses related to the assimilation of training load in athletes.

Thus, in this study, we aimed to investigate whether there are changes in microvascular (i.e., SmO_2_) responses of exercising and non‐exercising muscles using NIRS technology during an isometric test pre‐ and post‐acute an exercise protocol. Considering these physiological mechanisms and previous findings, we hypothesized that: (i) microvascular responses would affect all muscles assessed and not only the exercised muscle, taking into account the previous results of Yogev et al. (2022), and (ii) the exercised limb would show greater changes in microvascular responses between pre‐ and post‐exercise than the contralateral non‐exercised leg.

## 2 MATERIALS AND METHODS

### 2.1 Participants

This study included 14 male participants, but due to technical problems with signals from two participants, the data analyses only considered 12 participants (Table 1). Inclusion criteria included no history of smoking, aged between 18 and 40 years old, having no history of chronic diseases or recent surgery, and should be regularly enrolled in physical exercise of low‐ to moderate‐intensity sessions lasting at least 1 h per week. Participants were informed both verbally and in writing about the procedures, potential risks, and benefits of the tests, and they provided written consent before the commencement of the study. The study procedures complied with the Declaration of Helsinki and were approved by the University of Valencia ethics committee (IRB #2626957).

**TABLE 1:** Mean and standard deviation of the participants' characteristics.

Characteristics	*n* = 12
Age (years)	25 ± 6
Height (m)	1.75 ± 0.07
Body mass (kg)	71.9 ± 7.8
Body mass index (kg·(m^2^)^−1^)	23.58 ± 2.22
IPAQ (a. u.)	75.41 ± 82.13
Preferred thigh skinfold (mm)	18.5 ± 8.5
Non‐preferred thigh skinfold (mm)	19.1 ± 8.9
Triceps skinfold (mm)	12.8 ± 7.4

Abbreviations: IPAQ, International Physical Activity Questionnaire.

### 2.2 Experimental design

**FIGURE 1:**
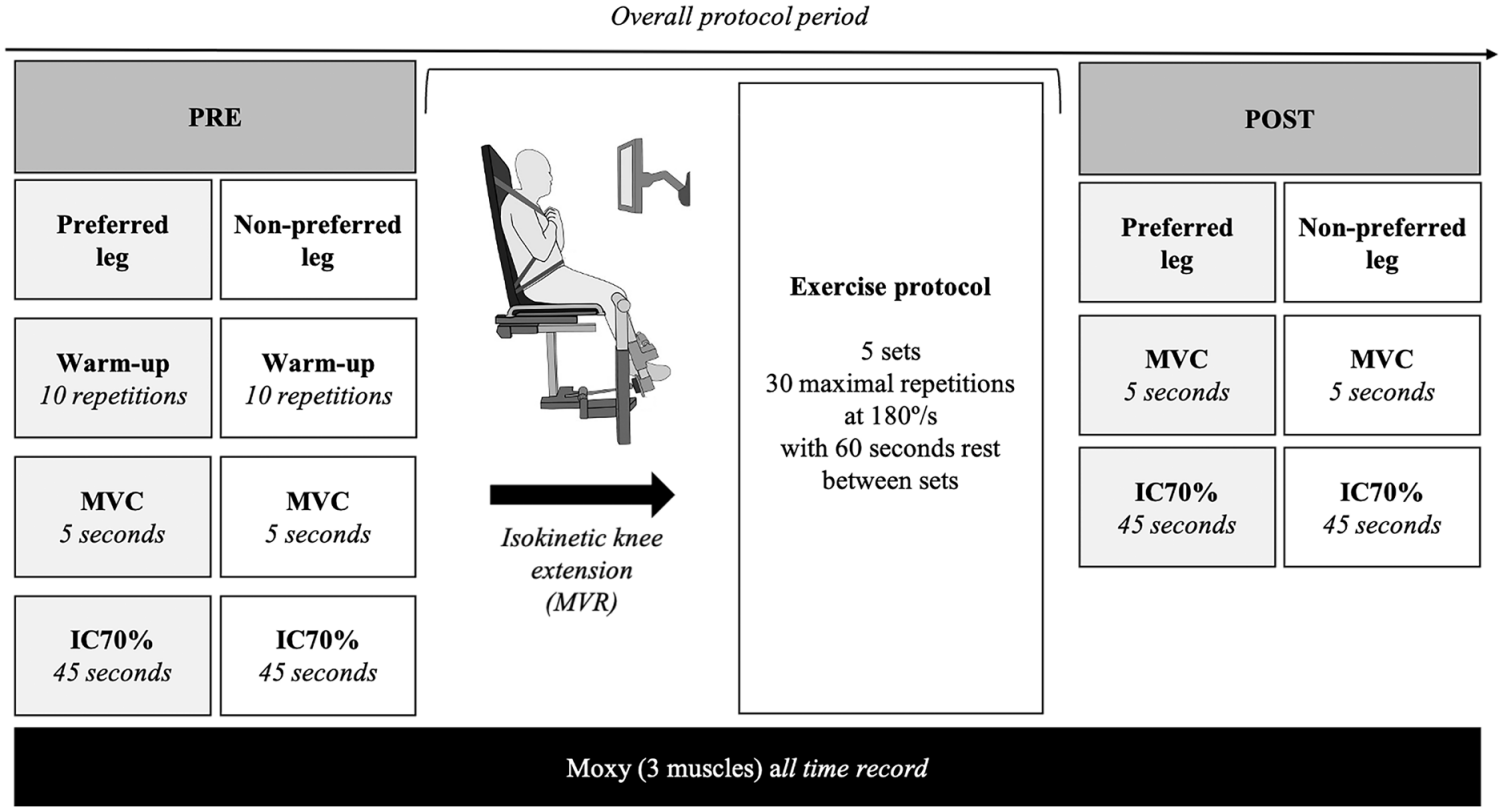
Flow diagram of the exercise protocol. IC70%, isometric contraction at 70% of the MVC; MVC, maximal voluntary contraction; MVR, maximal voluntary repetition.

We conducted a cross‐sectional, case–control study including healthy adult male participants who visited the laboratory once for all tests. In the 24 h prior to data collection, they were instructed to sleep at least 7 h at night and avoid consuming alcohol or any other stimulants, as well as performing physical exercise. Participants came to the laboratory to be assessed with regard to unilateral isometric knee extension torque using an isokinetic dynamometer (Biodex Multi‐joint System Pro 3, Shirley, NY, USA) and SmO_2_ using a Moxy Monitor (Fortiori Design LLC, Minneapolis, USA) before and after completing a knee extensor exercise protocol. The protocol included an exercise of 5 sets (Rawson, 2010) to mimic a training session, maximal voluntary contractions (MVC) before and after the sets to evaluate the changes in torque application, and an isometric contraction of 45 s to assess the changes in SmO_2_ induced by the exercise sets. Participants initially completed a progressive warm‐up for the non‐preferred leg (10 knee extensions without load) to prepare for the subsequent evaluations for this leg. After the warm‐up, the non‐preferred leg was evaluated regarding the torque output during three MVCs lasting 5 s each, and completed one submaximal isometric contraction lasting 45 s at an intensity corresponding to 70% of the MVC (IC70%) with the knee at 60° extension. After 3 min (necessary time to modify the arm position of the isokinetic dynamometer), participants started the exercise protocol again with their preferred leg (i.e., warm up, three MVC and one IC70%), and then, the exercise protocol consisted of 5 sets of 30 repetitions of concentric knee extensions performed at 180°/s with a 60‐s rest between sets (Rawson, 2010). The five sets were performed only with the preferred leg, which was identified by individual preference to kick a ball aiming at a target (van Melick et al., 2017). After 3 min, the preferred leg was evaluated again with regard to the isometric knee extension torque and muscle oxygenation during one repetition of IC70%, following which the non‐preferred leg was tested again. A schematic representation of our experimental design is depicted in Figure 1.

### 2.3 Data acquisition and analyses

Torque data (Nm) was sampled at 100 Hz by the isokinetic dynamometer and it was normalized to individual body mass (Nm·kg^−1^). The peak torque for each MVC was extracted and averaged for each leg (preferred and non‐preferred leg) and moment (pre‐ and post‐acute exercise protocol). The accumulative torque was considered as the sum of the peak torque from all repetitions during the acute exercise protocol (Rawson, 2010). The mean torque and the coefficient of variation were obtained during the isometric contractions (IC70%).

The SmO_2_ (%) was sampled at 2 Hz using NIRS technology (Moxy Monitor, Fortiori Design LLC, Minneapolis, USA). The NIRS devices were placed over the muscle belly of both vastus lateralis (at 2/3 of the distance between the line from the anterior superior iliac spine to the lateral side of the patella), and triceps brachii preferred (at 1/2 on the line between the posterior crista of the acromion and the olecranon at two finger widths medial to the line). These locations were based on the recommendations for placement of surface electromyography electrodes (Hermens et al., 1999) and agreed with a previous study (Batterson et al., 2023). The variation in SmO_2_ (∆SmO_2_) was assessed during the performance of IC70% considering a sliding window of 10 s (Sendra‐Pérez et al., 2023). In addition, minimum ∆SmO_2_ (∆SmO_2Min_), mean (∆SmO_2Mean_), slope of the SmO_2_ (Slope), and slope in the first 10 s of reoxygenation after IC70% (Slope10) were quantified during IC70%.

### 2.4 Statistical analyses

Statistical analysis was performed using Python (Anaconda Navigator 2.4) and RStudio (version 2023.12.0+369). Data distribution normality was checked using the Shapiro–Wilk test. Mean and standard deviation were used to describe variables showing a normal distribution. Otherwise, data are described considering the median and the interquartile range (IQR). The normality of the data was confirmed using the Shapiro–Wilk test (*p* > 0.05). Peak torque in the MCV, mean torque in the IC70%, and the coefficient of variation in the IC70% were compared pre‐ versus post‐exercise for each leg using paired *t*‐tests. A one‐way ANOVA was used to verify differences between series of the exercise protocol in peak torque and accumulative torque, with a paired *t*‐test with Bonferroni post hoc correction. One‐dimensional statistical parametric mapping (SPM) was used to compare the ∆SmO_2_ signals pre‐ versus post‐IC70% (being 0% at the beginning of the IC70% and 100% at its end). Differences in SmO_2_ between muscles (i.e., both vastus lateralis and triceps brachii) were verified using SPM applying a one‐way repeated‐measures ANOVA with a paired *t*‐test with Bonferroni post hoc correction (*p* = 0.017). Then, differences between pre‐ and post‐exercise were compared between limbs in vastus lateralis (i.e., exercising and non‐exercising leg) comparing the time series signals of ∆SmO_2_, using a paired *t*‐test. The pre‐ versus post‐exercise comparison of variables obtained from the signal (∆SmO_2Min_, ∆SmO_2Mean_, Slope and Slope10) was performed using the Wilcoxon test. For the significant differences, Cohen's effect sizes (ES) were computed and classified as small (ES 0.2–0.5), moderate (ES 0.5–0.8), or large (ES > 0.8) (Cohen, 1988). All statistical tests considered an alpha set at 5%.

## 3 RESULTS

### 3.1 Torque responses

Peak torque determined in the MVC did not differ pre‐ versus post‐exercise in the preferred (2.2 ± 0.6 Nm·kg^−1^ vs. 2.2 ± 0.4 Nm·kg^−1^, *p* = 0.98) and non‐preferred leg (2.3 ± 0.6 Nm·kg^−1^vs. 2.4 ± 0.5 Nm·kg^−1^, *p* = 0.68).

Although the mean torque during the IC70% was similar before and after exercise for the preferred (1.7 ± 0.4 Nm·kg^−1^ vs. 1.6 ± 0.3 Nm·kg^−1^, *p* = 0.69) and non‐preferred limb (1.6 ± 0.4 Nm·kg^−1^ vs. 1.7 ± 0.4 Nm·kg^−1^, *p* = 0.24), the coefficient of variation was lower after exercise, with a large effect size for the preferred (13.6 ± 2.6% vs. 9.4 ± 3.0%, *p* < 0.01 and ES = 1.5) and non‐preferred lower limb (14.9 ± 3.4% vs. 11.4 ± 2.8%, *p* = 0.01 and ES = 1.1).

**FIGURE 2:**
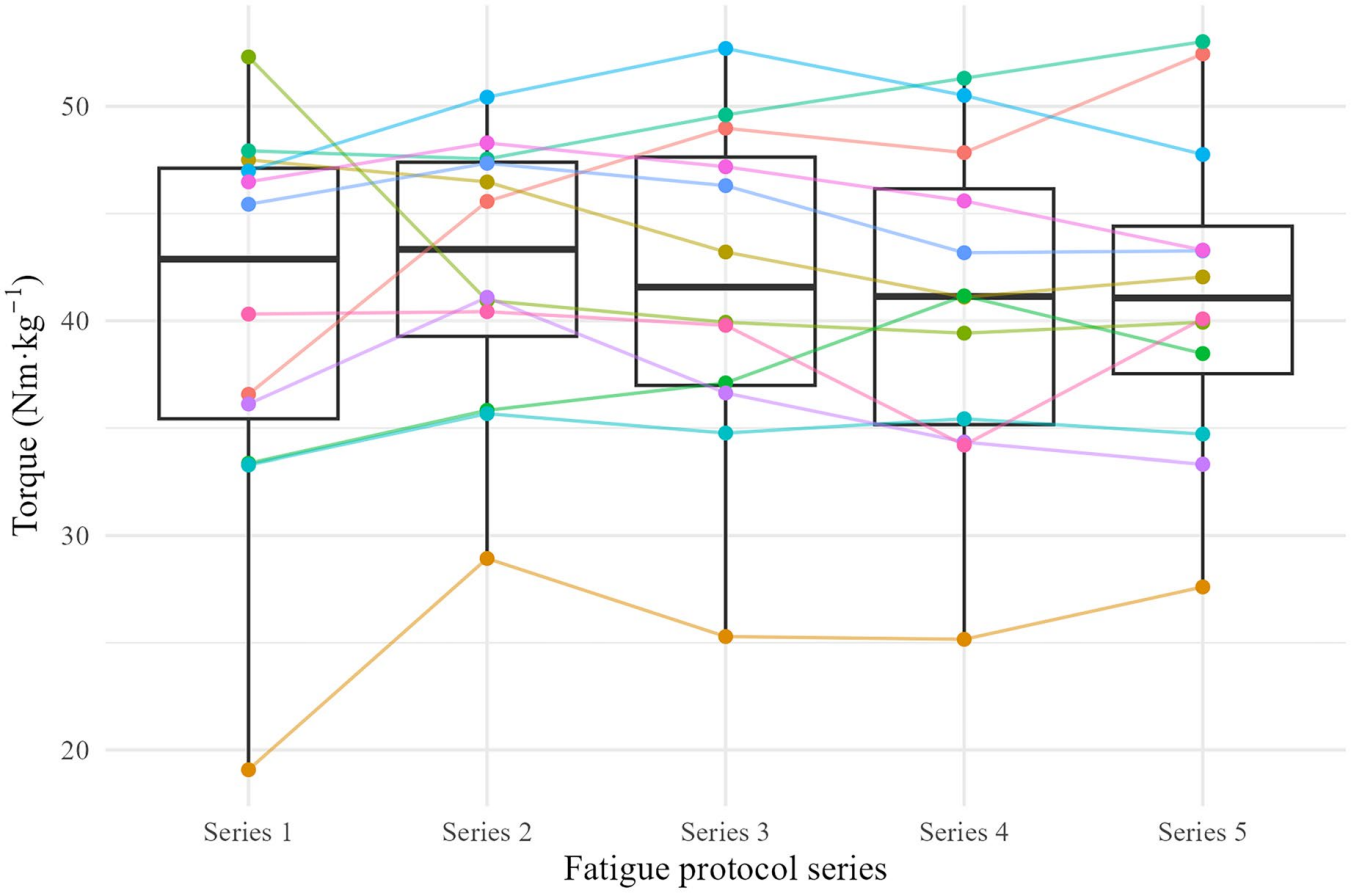
Accumulated torque (sum of the 30 peak torques) of the 5 series exercise protocol. The box shows the accumulated torque and the points of each participant in each series.

During the 5 series of 30 repetitions, the accumulated torque did not differ between the series (*p* = 0.98, Figure 2).

### 3.2 Muscle oxygenation: Exercise responses

**FIGURE 3:**
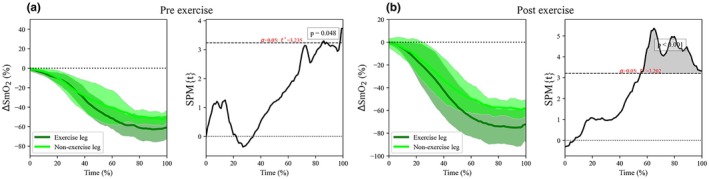
Statistical parametric mapping comparison of variation in the muscle oxygen saturation (∆SmO_2_) in both vastus lateralis during the IC70% exercise. Left column shows the pre‐exercise and the right columns shows the post‐exercise responses from vastus lateralis. The left figure from each panel shows the averaged time series of the ∆SmO_2_. The shaded area represents the standard deviation of the ∆SmO_2_. The right figure from each panel depicts the paired *t*‐test outcomes of muscle oxygen saturation activity of the control condition compared to the ∆SmO_2_. A significant effect is identified when the black continuous line exceeds the horizontal dotted line placed on the top and bottom of the figures.

The ∆SmO_2_ during IC70% exercise elicited differences between exercised and non‐exercised legs in the pre‐ (*p* value = 0.048) (Figure 3a) and post‐measurements (*p* value < 0.001) (Figure 3b). Post‐exercise differences were identified from around 50% of the exercise time to the end of the trial, while in the pre‐exercise differences appeared only in the very last seconds of the IC70%. Additional parameters related to changes in SmO_2_ during IC70% exercise showed differences between limbs (exercised vs. non‐exercised) in both moments (pre‐ and post‐exercise), as described in the Table 2.

**TABLE 2:** SmO_2_ outcomes in the IC70%. Median (IQR).

Variables	Pre‐exercise			Post‐exercise		
	Exercise leg	Non‐exercise leg	*p* Value (ES)	Exercise leg	Non‐exercise leg	*p* Value (ES)
∆SmO_2Min_ (%)	−54 (−58, −51)	−67 (−73, −58)	**0.01 (1.0)**	−60 (−63, −58)	−83 (−85, −76)	**<0.001 (1.3)**
∆SmO_2Mean_ (%)	−32 (−38, −25)	−36 (−41, −34)	0.1 (0.7)	−35 (−44, −29)	−46 (−60, −37)	0.05 (0.9)
Slope (%·sec^−1^)	−0.76 (−0.86, −0.65)	−1.23 (−1.67, −0.96)	**0.01 (1.0)**	−0.83 (−0.99, −0.79)	−1.30 (−1.49, −1.17)	**0.01 (1.1)**
Slope10 (%·sec^−1^)	1.01 (0.34, 1.52)	1.06 (0.93, 1.23)	0.7 (0.2)	1.09 (0.38, 1.39)	1.24 (0.29, 1.63)	0.8 (0.01)

*Note*: Bold values indicate statistically significant differences (*p* 〈 0.05).

Abbreviations: ∆SmO_2Mean_, mean of the variation of the muscle oxygen saturation; ∆SmO_2Min_, minimum of the variation of the muscle oxygen saturation; Slope10, slope in the first 10‐s of reoxygenation.

### 3.3 Muscles oxygenation: Individual muscle responses during IC70% exercise performed by the exercising vastus lateralis

**FIGURE 4:**
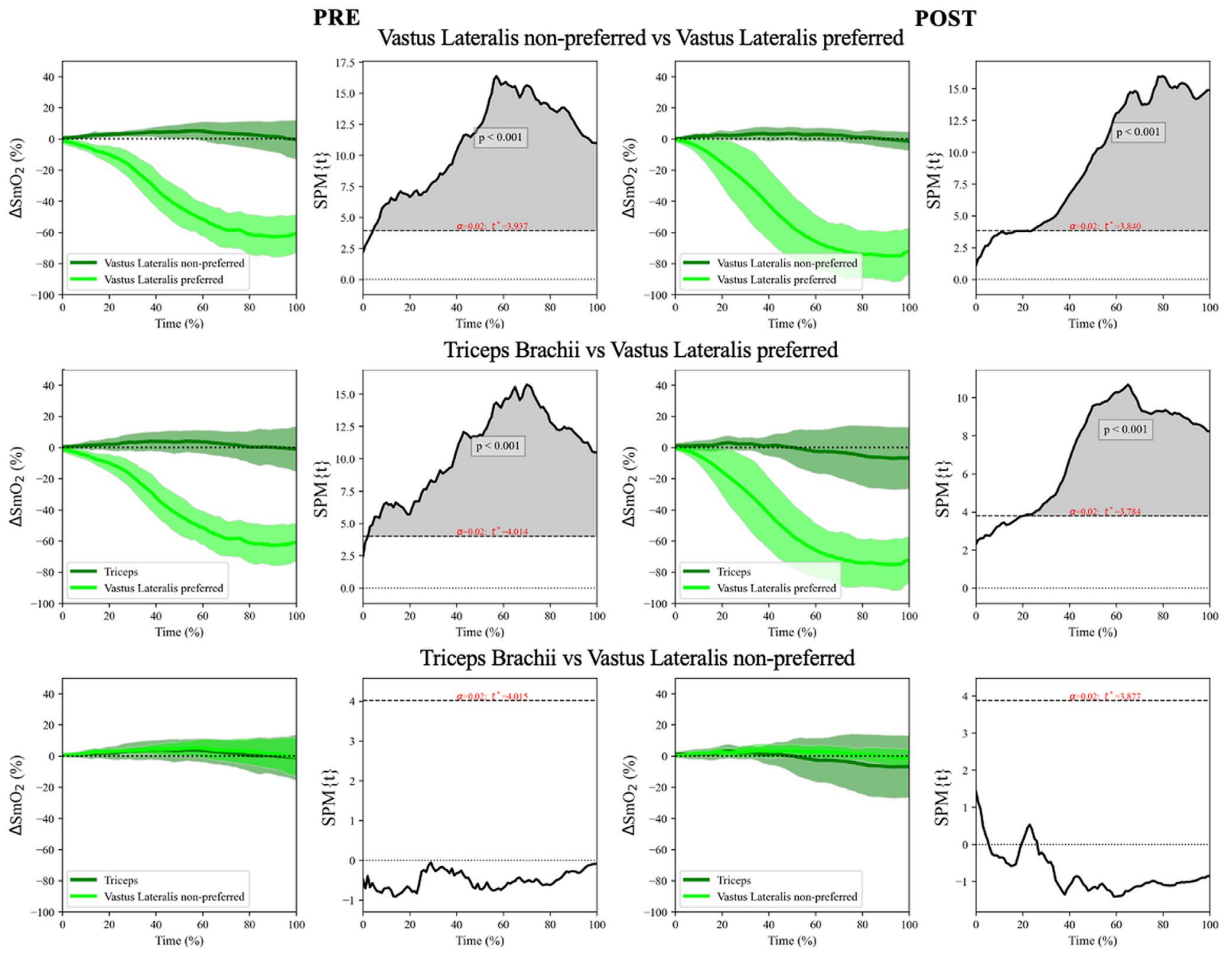
Statistical parametric mapping analysis comparing variation of muscle oxygen saturation (∆SmO_2_) in three locations (triceps brachii on the preferred limb and vastus lateralis on both limbs). Left column shows the pre‐exercise and the right columns shows the post‐exercise responses from vastus lateralis. The left figure from each panel shows the averaged time series of the ∆SmO_2_. The shaded area represents the standard deviation of the ∆SmO_2_. The right figure from each panel depicts the paired *t*‐test outcomes of muscle oxygen saturation activity of the control condition compared to the ∆SmO_2_. A significant effect is identified when the black continuous line exceeds the horizontal dotted line placed on the top and bottom of the figures.

∆SmO_2_ in the exercising vastus lateralis muscle consistently decreased while the ∆SmO_2_ in the other locations (triceps and non‐exercising vastus lateralis) did not differ between pre‐ and post‐IC70% exercise of the preferred vastus lateralis (difference between vastus lateralis in the exercising leg and the other locations; *p* value < 0.001, Figure 4). Additionally, there were no significant differences in ∆SmO_2_ between triceps brachii and vastus lateralis non‐exercising during the IC70% of the exercising vastus lateralis (*p* value > 0.05, Figure 4).

## 4 DISCUSSION

To determine different acute changes in microvascular responses during an isometric test using NIRS technology (SmO_2_) due to the exercise protocol (i.e., 5 sets of 30 repetitions), we analyzed differences in responses to exercised and non‐exercised legs in the vastus lateralis. SmO_2_ responses were affected by the exercise protocol, and significant differences were found between the exercised and non‐exercised legs. Our hypotheses regarding microvascular responses being influenced by exercise and allowing the identification of exercise‐dependent responses in the exercised muscles using an isometric test were confirmed. Although this experiment has inherent limitations, we believe that our experimental design could serve as a useful protocol to assess acute microvascular responses to exercise.

Our findings are in line with recent studies showing that NIRS‐derived SmO_2_ can detect subtle changes in oxygen extraction and delivery during occlusion of different durations (Iannetta et al., 2019; Rosenberry et al., 2018). It is already known that exercise modifies oxygen extraction and, in that sense, SmO_2_. Although our protocol involved a heavy workload, we did not observe differences in torque values considering that the peak torque in the MVC did not differ pre‐ versus post‐ the IC70%, in the same way as accumulative torque during the series did not drop (Rawson, 2010), even though we found a lower coefficient of variation in the preferred and non‐preferred lower limb. These findings may be due to a greater recruitment of the motor units in vastus lateralis to maintain the force as the muscle works, following the size principle (Murrant et al., 2021; Riley et al., 2008) as larger motor units are more consistent and less variable in force production at high exercise intensities than smaller units (Yao, 2004). Therefore, we can consider that our protocol mimics a training session with a high volume of load without inducing acute fatigue.

In addition, in the IC70% test, we found different responses for SmO_2_. Although the mean torque in the IC70% did not differ between the legs (pre‐ and post‐exercise protocol), different SmO_2_ responses were observed for exercised and non‐exercised muscles, as well as in the oxygen kinetics during the IC70%. These results are consistent with a previous study where peak torque in MVC did not differ between normoxia and hypoxia conditions, but significant differences were found in the vastus lateralis oxygenation (Dominelli et al., 2024). Our results suggest that changes in SmO_2_ might be useful for assessing microvascular responses and might be related to the exercise or task performed. While some studies have investigated associations between physiological parameters, such as VO_2Max_ and peak power output, with muscle oxidative capacity, using NIRS technology during occlusions (Beever et al., 2020; Possamai et al., 2023), few studies have comprehensively addressed acute exercise responses using this technology in protocols of lower complexity.

The exercise‐dependent responses are supported by our results showing that IC70% elicited changes in microvascular responses in the exercised vastus lateralis compared to the non‐exercised muscles (i.e., triceps brachii and vastus lateralis of the contralateral leg). This pattern was repeated before and after acute exercise (i.e., the non‐exercising leg did not show reduced oxygenation during the IC70%), despite muscle oxygenation being correlated with pulmonary oxygen uptake during knee extension exercise at 40% of MVC (Bringard & Perrey, 2004). However, in other effort types (e.g., graded exercise testing), various muscles showed similar oxygen kinetics, and O_2_ extraction by redistribution of blood flow continues to working muscles in the severe domain (Yogev et al., 2022). This study, however, did not observe this redistribution from the other muscles (i.e., triceps brachii and vastus lateralis), which may be due to the fact that the IC70% test was performed by one individual muscle group. In this context, previous literature has identified various physiological mechanisms in evaluating microvascular responses with dynamic or isometric exercise (Krüger et al., 2019). These differences may account for the variations in outcomes observed in our study. Moreover, NIRS technology allows for evaluating the oxidative capacity in specific muscular regions (Perrey et al., 2024), potentially showing local responses and preventing overwork injuries in specific regions.

Our study has some experimental considerations that should be mentioned. First, NIRS technology was used with participants having different adipose tissue thicknesses, although all had skinfolds of less than 25 mm (van der Zwaard et al., 2016). Second, it is important to consider that other NIRS devices can be more reliable, but the Moxy monitor is one of the most applicable in the field. In this sense, it is important to understand that the SmO_2_ of the Moxy Monitor is not comparable with other devices (McManus et al., 2018), which could result in different absolute values, although we expect similar relative responses. In future research, this test should be undertaken using different workloads and fatigue levels, with an additional measure designed to monitor muscle fatigue, such as EMG.

This study has important practical applications, as it proposes an isometric test for assessing the acute effects of exercise through muscle oxygenation in the exercised muscle, demonstrating that oxygenation remains unchanged in other muscles. The isometric test may be used before and after training or at different times of the season, to assess the training load. However, further studies should explore whether it can differentiate the responses depending on the level of exercise, and also by protocols more applicable to the field (e.g., isometric squats without using an isokinetic dynamometer).

## 5 CONCLUSION

We concluded that acute responses to exercise determined using portable near‐infrared spectroscopy during an isometric test may differ from torque responses. In addition, the isometric test does not increase muscle O_2_ extraction, with no flow redistribution from other muscles to the one performing the task. Our results encourage more research into validating this test for monitoring training loads.

## ETHICS STATEMENT

The Ethics Committee of the University of Valencia guaranteed the ethical approval to carry out this project with the document registered under number IRB #2626957.

## Data Availability

The datasets generated during and/or analyzed in the current study are not publicly available due to participant confidentiality, but they can be obtained from the corresponding author upon reasonable request.
